# Location-Based Resource Allocation in Ultra-Dense Network with Clustering †

**DOI:** 10.3390/s21124022

**Published:** 2021-06-10

**Authors:** Seong-Jung Kim, Jeong-Gon Kim

**Affiliations:** Department of Electronic Engineering, Korea Polytechnic University, Siheung 15073, Korea; ksj322@kpu.ac.kr

**Keywords:** ultra-dense network, clustering, *k*-means, resource allocation, joint CoMP

## Abstract

With the rapid deployment of present-day mobile communication systems, user traffic requirements have increased tremendously. An ultra-dense network is a configuration in which the density of small base stations is greater than or equal to that of the user equipment. Ultra-dense networks are considered as the key technology for 5th generation networks as they can improve the link quality and increase the system capacity. However, in an ultra-dense network, small base stations are densely positioned, so one user equipment may receive signals from two or more small base stations. This may cause a severe inter-cell interference problem. In this study, we considered a coordinated multi-point scenario, a cooperative technology between base stations to alleviate the interference. In addition, to suppress the occurrence of severe interference at the cell edges, link formation was carried out by considering the degree of cell load for each cluster. After the formation of links between all the base stations and user equipment, a subcarrier allocation procedure was performed. The subcarrier allocation method used in this study was based on the location of base stations with clustering to improve the data rate and reduce the interference between the clusters. Power allocation was based on the channel gain between the base station and user equipment. Simulation results showed that the proposed scheme delivered a higher sum rate than the other resource allocation methods reported previously for various types of user equipment.

## 1. Introduction

With the deployment of 5th generation (5G) networks, the use of internet-enabled devices and mobile devices has increased rapidly. The accompanying rapid development of mobile communication technology has tremendously increased user traffic requirements. To meet these requirements, 5G systems incorporate a novel ultra-dense network (UDN) [1]. A UDN is a configuration in which the density of small base stations (SBSs) is greater than or equal to that of the user equipment (UE) [2]. This arrangement densely populates the service areas with SBSs having small cell coverage as that observed in picocells and femtocells. The UDN configuration offers advantages such as increased maximum data rate, expanded cell coverage, flexible deployment of base stations (BSs), and improved link quality [3]. However, it suffers from serious inter-cell interference, which is caused by its high SBS density. A decrease in the distance between the adjacent cells increases the interference as the coverage can be overlapped very often [4]. Hence, any UE located at the edge of a cell receives multiple signals from two or more SBSs at the same time. This may cause an increase in the inter-cell interference during the processing of the UE and a significant decrease in the network capacity.

To solve this problem, the coordinated multi-point (CoMP) method, which involves a cooperative effect between the BSs, has been proposed [5]. The joint CoMP method involves multiple BSs that cooperate to collectively send the same data to the user [6]. Using this method, a UE located at the edge of a cell can receive signals from different BSs through multiple-in multiple-out antennas, mitigating the severe interference and achieving a higher data transmission rate. In addition, in this method, a BS can limit the number of UEs to the maximum number of subcarriers. Thus, this method facilitates the reduction of interference by allocating an orthogonal subcarrier to each UE to prevent the allocation of the same subcarrier to the adjacent UEs served by different BSs. However, the interference generated in the network is still high because of the high density of the BSs and UEs in the UDN environment. When the same subcarrier as the adjacent UE is used, the distance between the UE and BS reduces, which increases the interference and reduces the transmission rate. Therefore, the development of an appropriate resource allocation scheme is imperative to solve this problem.

The *k*-means algorithm, which a well-known clustering technique, can be applied to many practical problems owing to its simplicity and low computational complexity [7]. Various approaches have been proposed for clustering-based resource allocation in a UDN environment [8,9,10]. In a previous study, a clustering-based resource allocation scheme was used to improve the system throughout [8]. The efficiency of the sub-channels was improved by employing a three-stage approach involving clustering, sub-channel allocation, and power allocation. However, it was assumed that the BS served only the UE. A resource allocation scheme based on a clustering technique using the *k*-means algorithm has also been proposed. In this approach, a cluster was formed between each SBS, and orthogonal subcarriers were allocated to the UEs in the cluster. Then, the number of clusters with the optimal performance was determined. This method could mitigate the interference occurring between the adjacent cells. However, the interference between the adjacent clusters existed, and as the number of clusters increased, the distance between the adjacent clusters decreased, so the interference increased, and the data rate decreased. In another study [10], the authors proposed a resource allocation scheme based on the location and cluster and tried to increase the sum rate by reducing the interference between the adjacent clusters. In the present study, we modified the resource allocation scheme proposed in [10] and compared the performance of this scheme with those of the schemes proposed in [5,9].

In order to overcome the limitations of the resource allocation schemes discussed thus far, in this study, we proposed a clustering-based resource allocation scheme considering the location of the SBSs. First, a cluster between the SBSs was formed using the *k*-means algorithm. Then, resource allocation was performed, taking the SBS locations into account. This prevented the allocation of the same subcarrier to the adjacent SBSs in different clusters and the adjacent SBSs in the same cluster. After the subcarrier allocation, power allocation was performed so that differential power was allocated according to the channel gain. This subcarrier allocation scheme allocated subcarriers that were orthogonal to the adjacent UEs and increased the distance between the users of the same subcarrier to reduce the interference power. The proposed scheme was compared to the method of allocating frequencies with and without forming a cluster. Simulation results showed that the proposed scheme had a higher sum rate than the other resource allocation schemes.

This paper is organized as follows. Section 2 describes the system model used in this study in detail. In Section 3, the frequency resource allocation method is described, and in Section 4, the power allocation method is described. Section 5 discusses the simulation setup and the results obtained in this study. Section 6 concludes the paper.

## 2. System Model

This study assumed an orthogonal frequency-division multiplexing downlink scenario in the UDN environment. As a macro-BS and an SBS use different frequency bands, the interlayer interference was not considered. The randomness of the SBS location in the UDN environment was taken into account. The location of the SBS could be modeled as a point in the 2D or 3D Euclidean space according to a point process (PP) [4]. The most widely used PPs in wireless communication systems are the Poisson PP (PPP), Binomial PP (BPP), hard core PP (HCPP), and Poisson cluster process (PCP) [11]. The PPP is used to model or abstract a network composed of possibly an infinite number of nodes coexisting randomly and independently in a finite or infinite service area. In this study, we focused on checking the performance when the location of the UE changed continuously for each simulation. The SBS and UE were randomly positioned in the service area, and as mentioned earlier, the SBS density was higher than or equal to that of the UE in the UDN. In this situation, the high-density arrangement of the SBSs caused high cell coverage overlap. We assumed that all the BS and UE locations were known and that the data transmission was carried out by dividing the frequency bandwidth available in each BS into *C* orthogonal subcarriers. In this network, SBS b had a total of B instances and was represented as b = {1, 2, ···, B}. There were a total of U UEs represented as u = {1, 2, ···, U}. The received signal of UE u could be expressed by (1).

(1) $$y_{u}=\underset{b\in B_{u}}{\sum }\sqrt{p_{u}^{b}h_{u}^{b}}s_{u}+\underset{m\in B-B_{u}}{\sum }\sqrt{p^{m}h_{u}^{m}}s_{u^{\prime }}+n_{u}$$

where $B_{u}$ is the set of BSs serving UE u. b represents the BS in the set; m represents the BS that interfered with the UE u. $s_{u}$ is the transmitted signal of the UE u; $n_{u}$ is the added white Gaussian noise with $CN\left(0,\sigma^{2}\right)$. $p_{u}^{b}$ and $p_{⬚}^{m}$ denote the transmission power of the BSs providing service to the UE u and the interference power of the BSs providing the interference signal, respectively, and $h_{u}^{b}$ and $h_{u}^{m}$ represent the channel gain to the UE u. Here, we considered both large- and small-scale fading. The channel gain matrix can be expressed by (2).

(2) $$H=\left[\begin{array}{c} h_{1}^{1}\;\cdots \;h_{1}^{b}\;\cdots \;h_{1}^{B}\\ \vdots \;\;\;\;\;\;\;\;\;\;\;\vdots \;\;\;\;\;\;\;\;\;\;\;\vdots \\ h_{u}^{1}\;\cdots \;h_{u}^{b}\;\cdots \;h_{u}^{B}\\ \vdots \;\;\;\;\;\;\;\;\;\;\;\vdots \;\;\;\;\;\;\;\;\;\;\;\vdots \\ h_{U}^{1}\;\cdots \;h_{U}^{b}\;\cdots \;h_{U}^{B} \end{array}\right]$$

The three terms on the right side of (1) represent the useful signal, interference signal, and noise of the UE u, respectively. Therefore, the SINR of UE *u* can be defined as follows.

(3) $$SINR_{u}=\frac{\sum_{b\in B_{u}}^{⬚}p_{u}^{b}h_{u}^{b}}{\sum_{m\in B-B_{u}}^{⬚}p^{m}h_{u}^{m}+\sigma_{⬚}^{2}}$$

Then, based on (3), the data rate of the UE could be obtained using (4). The sum rate, which is the sum of the data rates $r_{u}$ of all UEs u, could be calculated from (4) using (5).

(4) $$r_{u}=Blog_{2}\left(1+SINR_{u}\right)$$

(5) $$R_{sum}=\overset{U}{\underset{u=1}{\sum }}r_{u}$$

**Figure 1:**
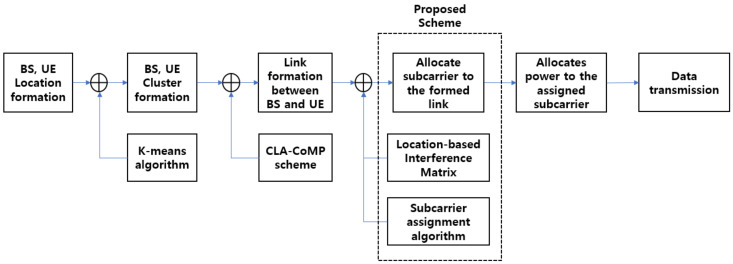
Proposed resource allocation scheme.

In (3), the interference signal of the same subcarrier could decrease the sum rate and was affected by the distance. The power of the interference signal of the same subcarrier increased as the distance and power increased. Therefore, the interference increased depending on the subcarrier allocated to the UE *u* $A_{s}$. By allocating the subcarriers that were orthogonal to each other between the adjacent UEs, the distance between the interference signals of the same subcarrier could be increased. Therefore, the power of the interference signal power decreased because of the increased distance, which increased the sum rate. In order to increase the sum rate, we used a resource allocation strategy. The resource allocation problem for maximizing the sum rate was defined as follows.

(6) $$\underset{\left\{s_{u}^{b},p_{u}^{b}\right\}}{\max}R_{sum}$$

(7) $$\overset{U}{\underset{u=1}{\sum }}s_{u}^{b}\leq S,\;\forall b\in B$$

(8) $$0\leq p_{u}^{b},\;\overset{U}{\underset{u=1}{\sum }}p_{u}^{b}\leq p_{max}^{b},\forall u\in U\;,\forall b\in B$$

where $s_{u}^{b}$ denotes the subcarrier allocated to the UE *u* by the BS *b*, and $s_{u}^{b}\in S$, $p_{max}^{b}$ represents the maximum transmission power of the BS *b*. Equation (7) indicates that the subcarriers could not be reused in each BS, and Equation (8) represents the constraints of the transmission power. The whole procedure for the proposed scheme is shown in Figure 1.

## 3. Resource Allocation Schemes

### 3.1. Cluster Formation

We chose the *k*-means algorithm from among the various clustering techniques widely used to perform SBS clustering. To divide the SBS into clusters, the number of clusters *k* and the dataset of the SBS were entered. Based on the input, the clusters of each SBS were provided as the outputs. First, the *k* initial center points were arbitrarily defined.

**Figure 2:**
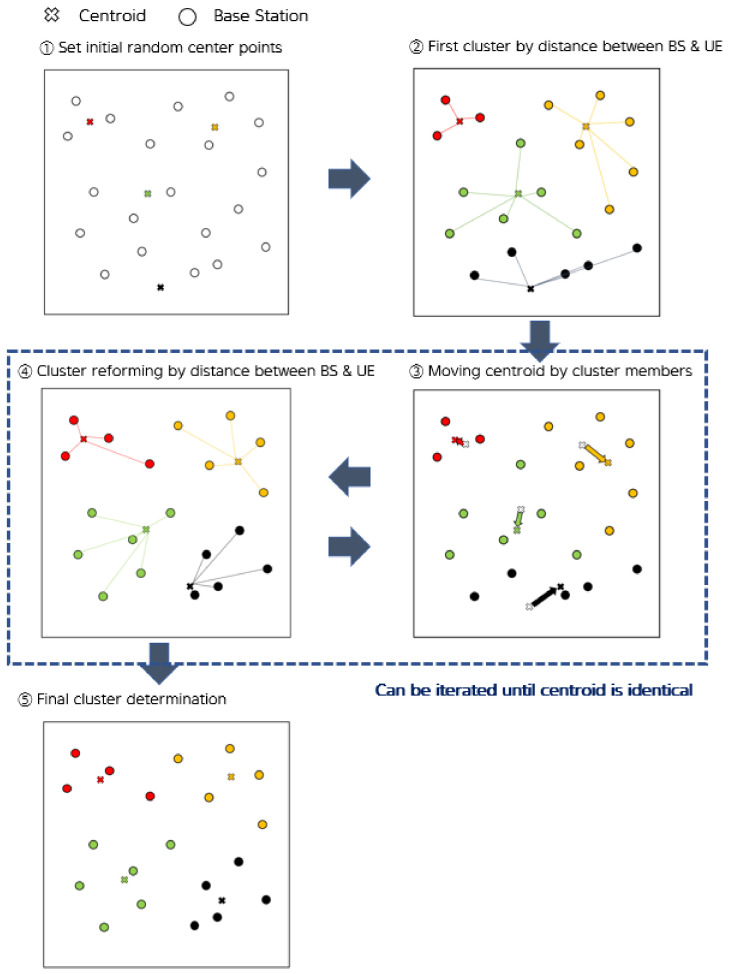
The cluster formation.

Then, each SBS was assigned to the cluster having the closest initial center point. The center point of each cluster was moved to the center of gravity of each cluster, and the SBSs were again assigned to the nearest center point. This process was continued until the center points converged. After the convergence of the centroids, each SBS was defined as a cluster of the closest centroids, and then, the UEs were associated with the closest SBS. Figure 2 illustrates this process of cluster formation and identifies different clusters with different colors.

### 3.2. Link Formation

**Figure 3:**
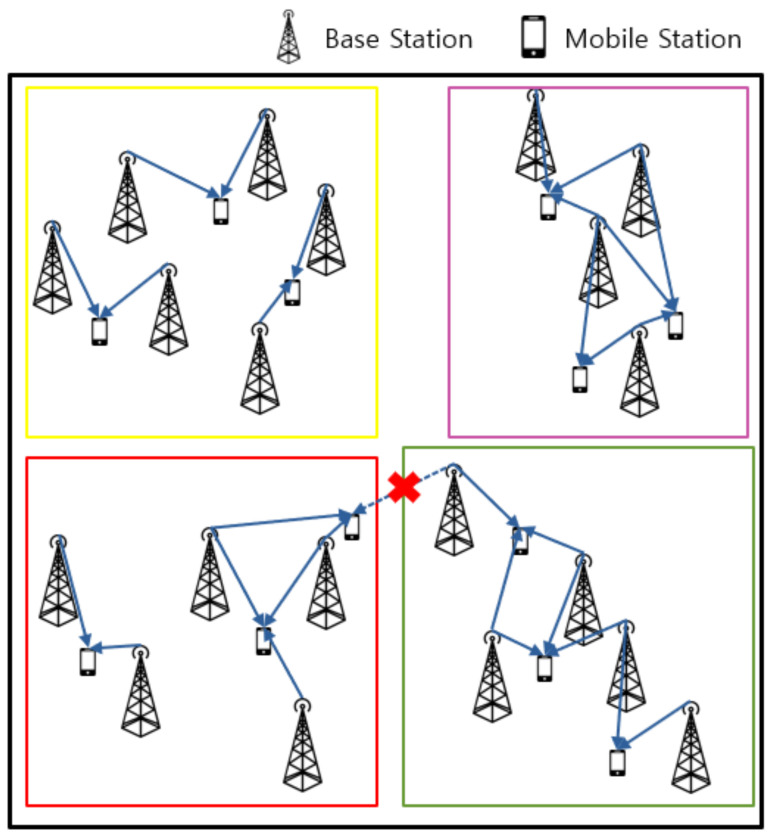
Link formation scheme.

Link formation was performed after the cluster formation of all the BSs and UEs. For the service link formation between the SBSs and UEs, the load level of the cell was considered. The radius of SBS coverage was assumed to be 15 m. Link formation was sequentially performed in the descending order of each UE’s channel gain in a cell coverage area. The link could not be provided from the SBS when the number of UEs was greater than the number of available subcarriers in the SBS. The link of a UE was released if it was already formed between the UE and SBS of an adjacent cluster. This is illustrated in Figure 3. In addition, when the number of UEs present in the cluster was greater than the number of subcarriers, the link of the lowest channel gain UE was released.

### 3.3. Proposed Subcarrier Allocation

Subcarrier allocation was performed after the formation of links between all the BSs and UEs. The subcarrier allocation algorithm was based on the distance between the BSs. This scheme also considered the distances of the BSs from the adjacent clusters, unlike conventional clustering-based subcarrier allocation schemes, which include only the BSs in a given cluster. In this method, different subcarriers were allocated to two UEs that were close to each other and belonged to different clusters. In this way, the interference between the adjacent clusters could be alleviated. The procedure for subcarrier allocation is described in the next section.

#### 3.3.1. Computing Location-Based Interference Matrix

First, we assumed that the locations of all the BSs in the network were already known. Given this assumption, the distance between the BSs could be calculated, and the distance between the b-th and B-th SBSs could be expressed as $d_{B}^{b}$. The distance between the SBSs was used to determine whether the SBSs were close to another cluster. The coverage of a BS was defined as dco; this distance was used to determine whether the coverage of one BS overlapped with that of another BS, and dco denotes the coverage of a BS, 15 m. From this information, we created a matrix G for the interference between the SBSs. The interference matrix G was formulated as in (9).

(9) $$G_{\;}=\left[\begin{array}{c} g_{1}^{1}\;\cdots \;g_{1}^{b}\;\cdots \;g_{1}^{B}\\ \vdots \;\;\;\;\;\;\;\;\;\;\;\vdots \;\;\;\;\;\;\;\;\;\;\;\vdots \\ g_{b}^{1}\;\cdots \;g_{b}^{b}\;\cdots \;g_{b}^{B}\\ \vdots \;\;\;\;\;\;\;\;\;\;\;\vdots \;\;\;\;\;\;\;\;\;\;\;\vdots \\ g_{B}^{1}\;\cdots \;g_{B}^{b}\;\cdots \;g_{B}^{B} \end{array}\right]⬚$$

where $g_{B}^{b}$ denotes the interference between the b-th and B-th SBSs. If the coverage of the two SBSs overlapped, $d_{B}^{b}$ was less than twice dco. If the SBSs were not the members of the same cluster, then $g_{B}^{b}$ was set at 1; otherwise, it was set at 0. The calculation process is described in detail in Algorithm 1.
**Algorithm 1** Process to Compute the G Matrix.**Input**: B is the number of BSs and $N_{i}^{⬚}\;\mathrm{and}\;N_{j}^{⬚}$ are the cluster number of the i-th and j-th SBSs. **Output**: interference between the i-th SBS and j-th SBS $g_{j}^{i}$.1:**for** i=1 to B2:      **for** j=1 to B3:          Calculate the distance between $d_{j}^{i}$ for all $i,j=\left\{1,2,\ldots ,B\right\}$;4:          **if** the cluster number $N_{i}^{⬚}\neq N_{j}^{⬚}$ and $d_{j}^{i}<30\;$5:            $g_{j}^{i}\leftarrow 1$6:       **else**7:            $g_{j}^{i}\leftarrow 0$

#### 3.3.2. Subcarrier Allocation

**Figure 4:**
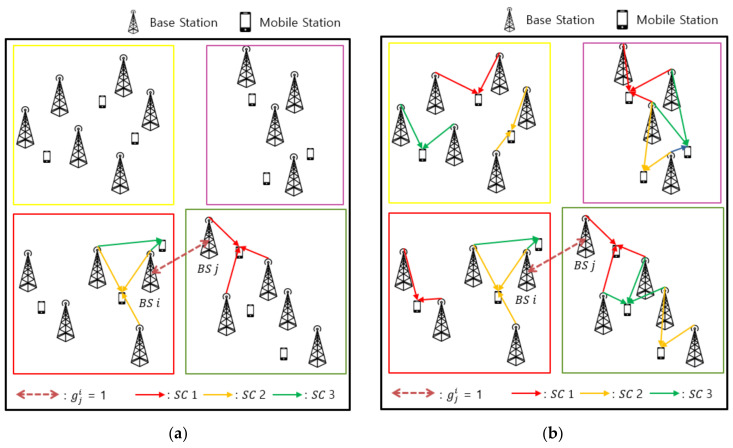
Subcarrier allocation: (**a**) between the BSs in the adjacent clusters and (**b**) between the BSs in the same cluster.

Based on (9), we allocated different subcarriers to $BS_{i}$ and $BS_{j}$ in the adjacent clusters, as shown in Figure 4a, to avoid the mutual interference between the two BSs. Then, the subcarriers were allocated to all the UEs common to each cluster on an orthogonal basis, as shown in Figure 4b. The subcarrier assignment process is described in detail in Algorithm 2. This was followed by Algorithm 1 and was iterated until all the subcarriers were allocated to the links determined in Section 3.2.
**Algorithm 2** Subcarrier Assignment Algorithm.**Input**: Subcarrier $s=\{s_{1},s_{2},\ldots s_{S}$), BS b={1,2,…B), UE u={1,2,…U). interference between the i-th and j-th BSs, $g_{j}^{i}$. **Output**: Subcarrier allocated from the BS b to the UE u $s_{u}^{b}$. **Initialization**: Subcarrier allocated to the UE u $A_{s}$.
1:**for** i=1 to B2:   **for** j=1 to B3:    **if** $g_{j}^{i}$ = 1, in i-th BS cluster and j-th BS are not use the s then4:     $A_{s}\;\leftarrow$ s served by the i-th BS5:   **for** k=1 to U6:    **if** UE k is not assigned subcarrier $A_{s}=0$ then7:     $A_{s}\;\leftarrow$ Unused subcarrier in UE k’s cluster s8:**for** m=1 to B9:   $s_{u}^{b}\leftarrow$ $A_{s}$ for all the BSs serving the UE u


## 4. Power Allocation Scheme

Power allocation was performed after the resource allocation between all the BSs and UEs. The maximum transmission power was allocated according to the channel gain values of the UEs served by the BS. High power was allocated to the users having high channel gain and was located at the center of a cell. On the other hand, low power was allocated to the users having low channel gain and was located at the edge of a cell. The power allocation improved the data rate of the entire network. The channel gain-based power allocation factor for each SBS-to-UE link is as follows:

(10) $$\alpha_{u}^{b}=\frac{h_{u}^{b}}{\sum_{m\in U_{b}}h_{m}^{b}}$$

where $\alpha_{u}^{b}$ denotes the power allocation factor for the UE *u* in the SBS *b*. The power allocation value was the product of the multiplies the calculated power allocation factor and by the maximum transmission power of the SBS and can be expressed as follows:

(11) $$p_{u}^{b}=\alpha_{u}^{b}P_{max}^{b}$$

Here, $p_{u}^{b}$ is the power allocation by the SBS *b* to the UE *u*, and $P_{max}^{b}$ is the maximum transmission power of the SBS *b*. After the power allocation of all the UEs, the performance of the proposed resource allocation was compared with those of the other previously reported approaches.

## 5. Simulation Results

**Figure 5:**
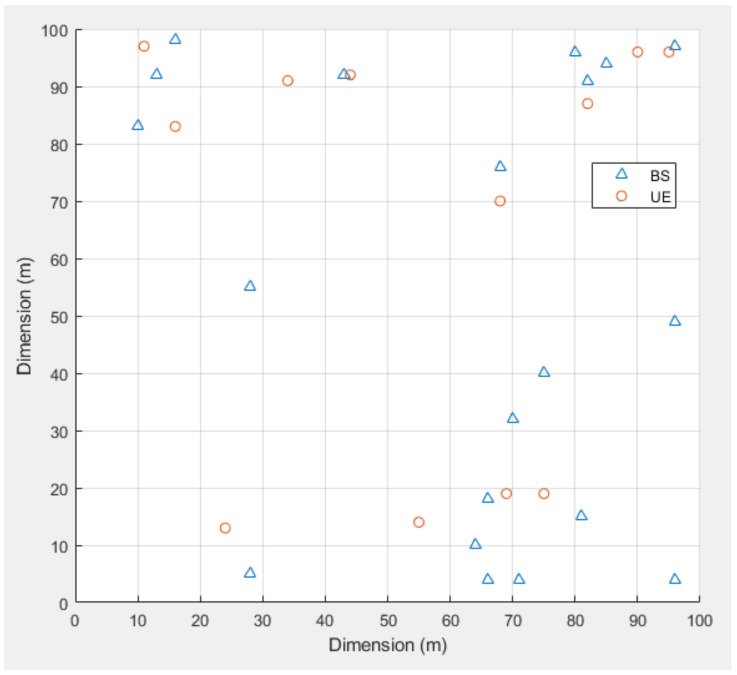
The simulation environment (100 m × 100 m, BS = 20, UE = 12).

We assumed a network with an orthogonal frequency division multiplexing (OFDM) downlink-based UDN environment for simulation purposes. The SBSs and UEs were randomly arranged in a 100 m × 100$\;\;m^{⬚}$ area as shown in Figure 5. The Monte Carlo method was used to verify the performance of the proposed method. The simulation was conducted 10,000 times, and the location of the UE changed randomly for each simulation in a fixed state. The simulation results showed the average performance for all the simulations. The parameters used in the simulation are summarized in Table 1. We used the data rate of the entire system for comparison purposes. The number of cluster center points, *k,* was set a 5 because the distance to the center point decreased when the cluster was formed, and the performance was compared with the case with 15 cluster center points.

**Figure 6:**
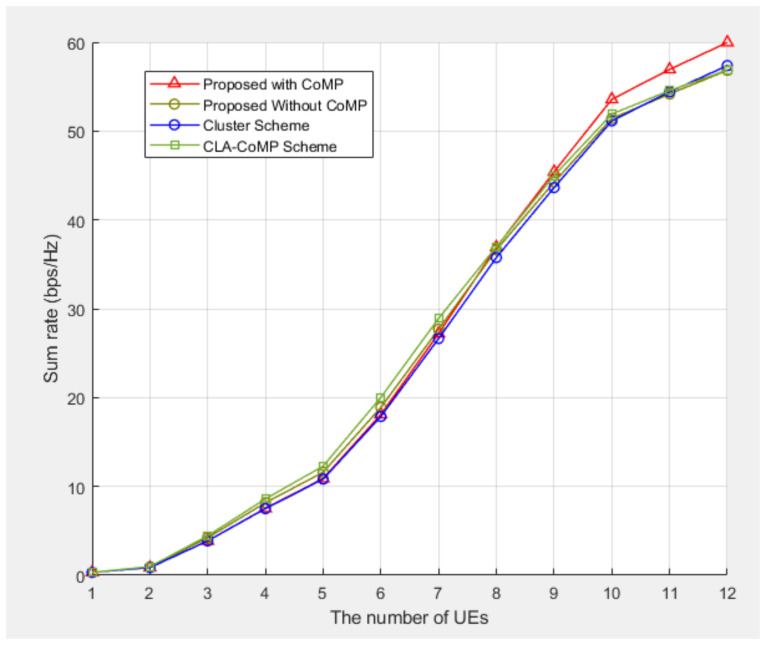
Sum rate performance comparison for five clusters.

Figure 6 shows the total sum rate for three resource allocation schemes: the cell load aware (CLA)-CoMP method [5], cluster method [9], proposed method with CoMP and without CoMP for five clusters. In the case of the without CoMP scenario, CoMP was excluded from the proposed scheme. Therefore, each UE received service only from the nearest BS. The sum rate performance of the proposed scheme improved as the number of UEs increased from 1 to 12. When the number of UEs was small, the probability of the interference due to the same subcarrier was small, and thus the difference obtained from the other schemes was small. As the CLA-CoMP method does not consider the subcarriers between the adjacent cells, and as the clustering method does not consider the subcarriers between the adjacent clusters, their sum rate performances were poorer than that of the proposed scheme. As the number of UEs increased, the difference between the performances of the methods increased gradually. This implies that the proposed scheme could effectively reduce the interference between the adjacent cells in a CoMP-based UDN environment. In addition, the sum rate improved when CoMP was taken into consideration as compared to the case when CoMP was not considered. Hence, the proposed scheme was more effective in increasing the system capacity when CoMP was applied.

**Figure 7:**
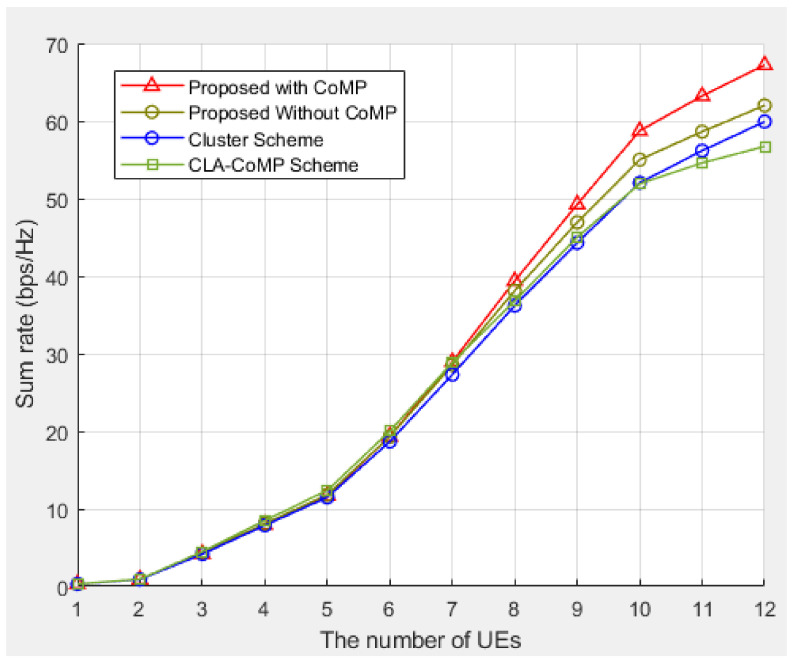
Sum rate performance of different methods for 15 clusters.

Figure 7 compares the sum rate performances of the three schemes for 15 clusters and shows that the proposed scheme exhibited the best performance among all the schemes investigated. With an increase in the number of clusters to 15, the proposed scheme reduced the interference between the clusters by using the location information of the BS and UE. The figure shows that the proposed scheme with more clusters exhibited even higher performance improvement than that shown in Figure 6. This indicates that the proposed scheme reduced the inter-cluster interference by avoiding duplicate subcarrier allocations to the BSs of the adjacent clusters in a clustered network. As shown in Figure 7, the proposed scheme improved the performance of the system, and the improvement in the performance was even higher when CoMP was applied.

**Figure 8:**
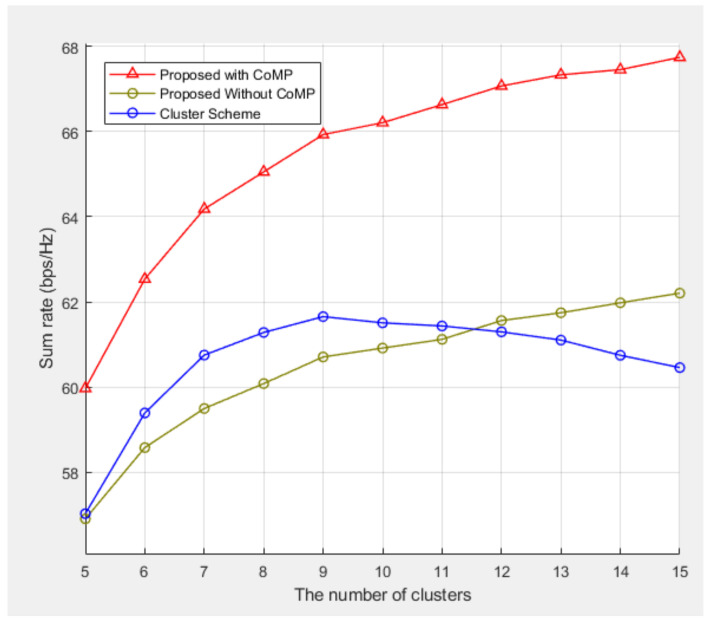
Sum rate performances of the three methods when the number of clusters was increased from 5 to 15.

Figure 8 compares the sum rate performances of the three schemes as the number of clusters was increased from 5 to 15 when there were 20 BSs and 12 UEs. With an increase in the number of clusters, the frequency reuse increased, and the sum rate increased gradually. When the number of clusters increased to 10, the sum rate performance of the cluster scheme deteriorated. The distance between the clusters shortened, and the interference between the clusters increased, and the sum rate performance deteriorated. However, as the proposed scheme allocated orthogonal subcarriers between the adjacent clusters, the performance improved even if the number of clusters continued to increase.

**Figure 9:**
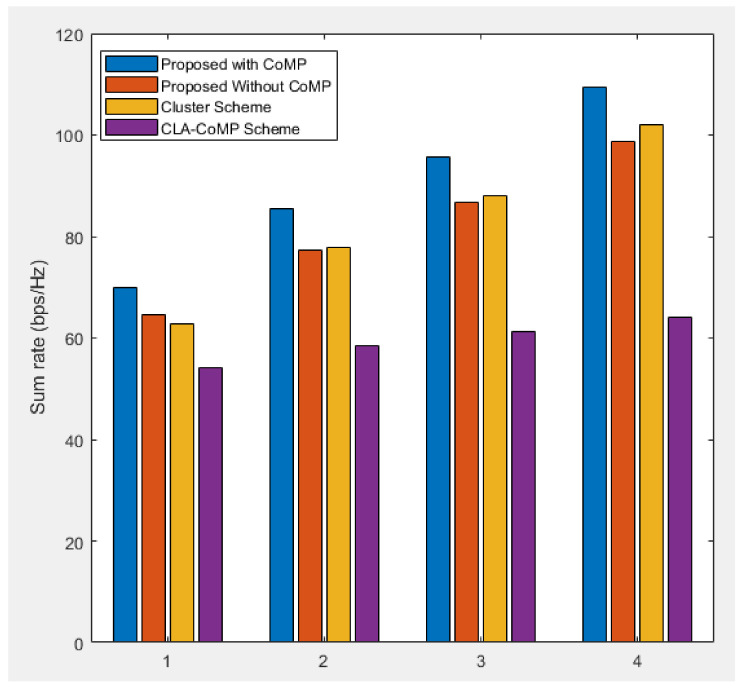
Sum rate performances of the three methods at different SBS and UE densities.

Figure 9 compares the sum rate performances of the three methods after increasing the density of the SBSs and UEs. In a 100 m × 100 m area, number 1 is BS 25 UE 12, number 2 is BS 30 UE 15, number 3 is BS 35 UE 17, and number 4 is BS 40 UE 20. Therefore, we compared the performances of the methods by increasing the SBS and UE density by 25%, 50%, 75% and 100%. With an increase in the SBS and UE density, interference between the adjacent cells increased, resulting in the deterioration of the sum rate performance. It can be observed from Figure 9 that the cluster scheme and proposed subcarrier allocation scheme improved the sum rate performance by reducing the interference power between the adjacent UEs, and the difference increased as the density increased.

## 6. Conclusions

In this study, we investigated radio resource management in a CoMP-based UDN environment. This environment is a key technology in 5G networks; it is used to meet the network traffic requirements. In a UDN environment, the distance between the adjacent cells is small, which increases the interference. This increase in interference reduces the data and sum rates of each UE. A resource allocation scheme based on a CoMP method to improve the data rate of an entire system was studied. To compare the performance of the proposed method with those of conventional schemes, link formation that considered cell load and clustering based on a *k*-means algorithm was incorporated for subcarrier allocation. The adjacent BSs were clustered through the k-means algorithm, and different subcarriers were allocated between the UEs in the cluster. The proposed resource allocation scheme reduced the interference between the adjacent clusters by allocating different subcarriers between the SBSs in the adjacent clusters. Simulation results showed that the proposed scheme improved the total sum rate by reducing the interference between the adjacent clusters very effectively. Moreover, it was verified that the proposed scheme increases the difference between the sum rate performances of the three methods as the number of clusters increased compared to the existing cluster-based frequency allocation scheme. The results showed that the proposed scheme effectively reduced the inter-cluster interference and, hence, increased the total sum rate. In the future, we will consider the initial central point of clustering to improve the sum rate performance. We will improve the sum rate performance by applying it to the UDN environment with an improved clustering scheme through the initial center point setting. In addition, we will carry out simulation studies to consider the randomness of the node location through the PPP.

## Figures and Tables

**Table 1:** The simulation parameters.

Parameter	Value
Number of SBSs	20
Number of UEs	12
Number of subcarriers	3
Service radius of the SBSs (m)	15
Path-loss exponent factor (u)	3.5
Variance of the noise	0.1
Maximum power of a SBS (dBm)	10
Number of clusters	5, 15

## Data Availability

Not applicable.
